# Advances in neoadjuvant immunotherapy for non-small cell lung cancer

**DOI:** 10.3389/fimmu.2026.1878102

**Published:** 2026-06-12

**Authors:** Xuehui Liu, Huasheng Li, Guangqi Dong, Yuyan Liu, Maobiao Ran, Dongbin Wang

**Affiliations:** 1 Department of Cardiothoracic Surgery, Tianjin Hospital, Tianjin University, Tianjin, China; 2 Department of Thoracic Surgery, Pengshui County People’s Hospital, Chongqing, China

**Keywords:** immune checkpoint inhibitors, major pathological response, neoadjuvant immunotherapy, non-small cell lung cancer, predictive biomarkers, tumor microenvironment

## Abstract

Non-small cell lung cancer (NSCLC) represents the majority of lung cancer cases and remains a leading cause of cancer-related mortality worldwide. Early-stage NSCLC is primarily treated with surgical resection; however, high recurrence and metastasis rates necessitate additional therapies. Emerging neoadjuvant immunotherapy, particularly immune checkpoint inhibitors (ICIs), has demonstrated efficacy in enhancing anti-tumor immune responses by restoring CD8^+^ T cell activity and promoting tumor-specific cytotoxicity. Preclinical studies indicate that combination strategies, including dual PD-1/CTLA-4 blockade, oncolytic viral therapy, and chemoimmunotherapy, synergistically improve survival and pathological response. Clinical trials have confirmed the safety and feasibility of these approaches, with neoadjuvant ICIs achieving major pathological response (MPR) and pathological complete response (pCR) in a substantial proportion of operable NSCLC patients. Predictive biomarkers, including PD-L1, tumor mutational burden (TMB), circulating tumor DNA (ctDNA), and immune profiling, may guide patient selection and optimize treatment outcomes. This review summarizes current evidence on the mechanisms, clinical efficacy, and biomarker-driven strategies for neoadjuvant immunotherapy in NSCLC, highlighting its potential to improve long-term survival and inform personalized therapeutic approaches.

## 1 Introduction

Although surgical resection constitutes the cornerstone of curative-intent treatment for early-stage non-small cell lung cancer (NSCLC), the persistent risks of locoregional recurrence and distant metastasis continue to compromise long-term survival outcomes (1, 2). Consequently, multimodal strategies incorporating perioperative systemic therapy have been actively pursued to augment surgical efficacy. While conventional adjuvant chemotherapy confers a modest 5% absolute improvement in 5-year overall survival, the advent of immune checkpoint inhibitors (ICIs) has fundamentally reshaped therapeutic paradigms across solid malignancies (3, 4). Neoadjuvant immunotherapy offers distinct immunological advantages over adjuvant approaches: administration while the primary tumor, regional lymphatic drainage, and tumor-associated antigenic reservoir remain intact enables optimal priming and clonal expansion of tumor-specific immunity (5, 6).

The presence of macroscopic disease provides abundant neoantigens for efficient capture by antigen-presenting cells, thereby facilitating robust activation of cytotoxic CD8^+^ T cells within the tumor microenvironment (7, 8). Conversely, post-resection antigenic depletion may attenuate the breadth and magnitude of systemic anti-tumor immune activation (5, 9). Accordingly, neoadjuvant checkpoint blockade may engender broader systemic immune responses, promote durable immune memory, and enhance eradication of occult micrometastases prior to definitive resection (10, 11). This review synthesizes current evidence regarding the mechanistic foundations, clinical efficacy, and biomarker-driven strategies for neoadjuvant immunotherapy in NSCLC, while critically addressing emerging challenges and future translational directions.

## 2 Basic principles of immunotherapy in NSCLC

### 2.1 Neoadjuvant immunotherapy

Contemporary immunotherapeutic strategies encompass cellular gene therapy, adoptive T-cell transfer, and oncolytic viral platforms (12). Among these, immune checkpoint inhibitors (ICIs) have emerged as pivotal agents that restore anti-tumor immunity by disrupting inhibitory signaling cascades governing T-cell priming and effector differentiation. The PD-1/PD-L1 axis attenuates TCR- and CD28-mediated signaling through recruitment of phosphatases such as SHP2, thereby suppressing T-cell proliferation, cytokine secretion, and cytotoxic function (13); pharmacological blockade of this pathway reinvigorates exhausted CD8^+^ T cells within the NSCLC microenvironment (14, 15). Conversely, CTLA-4 predominantly regulates early T-cell activation by competing with CD28 for B7 ligands on antigen-presenting cells within lymphoid compartments, thereby constraining co-stimulatory signaling and tumor-specific T-cell priming (16, 17); its inhibition expands tumor-reactive T-cell clones and diversifies the anti-tumor immune repertoire (18). Beyond acute signaling restoration, checkpoint blockade may partially reverse T-cell exhaustion and facilitate transcriptional-epigenetic reprogramming, providing mechanistic rationale for neoadjuvant application in NSCLC (19).

Preclinical murine models consistently demonstrate that neoadjuvant immune checkpoint blockade—whether via anti-CD25 therapy, anti-PD-1 monotherapy, or combined PD-1 blockade with CD137 stimulation—confers superior long-term survival and anti-metastatic immunity relative to adjuvant administration (20). These benefits correlate with enhanced infiltration and clonal expansion of tumor-specific CD8^+^ T cells, underscoring that an intact primary tumor provides an antigen-rich platform for effective T-cell priming (21, 22). Neoadjuvant intervention may augment T-cell receptor clonality, expand tumor-reactive effector subsets, and promote memory-like CD8^+^ T-cell generation capable of sustaining immune surveillance post-resection (23), a consideration of particular relevance in early-stage NSCLC, where occult micrometastases may persist despite complete surgical excision.

While PD-1 and CTLA-4 independently suppress T-cell activation, their combined blockade yields synergistic immunomodulatory effects in preclinical studies (24, 25). Dual inhibition may prove more efficacious than monotherapy: CTLA-4 blockade amplifies early T-cell priming and proliferation, whereas PD-1 blockade reinvigorates exhausted tumor-infiltrating lymphocytes; despite distinct mechanisms, these pathways converge to potentiate anti-tumor immunity (24, 26). Clinically, combined CTLA-4/PD-1 blockade has demonstrated efficacy in metastatic melanoma, advanced NSCLC, and microsatellite instability-high colorectal cancer (27, 28). Moreover, neoadjuvant immunotherapy improves outcomes in early-stage NSCLC models by attenuating pulmonary metastases and augmenting systemic anti-tumor immunity (29). Sequential or combined neoadjuvant PD-1/CTLA-4 blockade significantly prolongs survival versus monotherapy and elicits more robust anti-tumor responses than adjuvant or single-agent approaches (30, 31). Finally, platforms integrating PD-1 blockade with NF-κB receptor inhibition offer translational frameworks to elucidate synergistic mechanisms underlying novel combination immunotherapies in NSCLC (32).

### 2.2 Basic principles of chemoimmunotherapy

**Figure 1:**
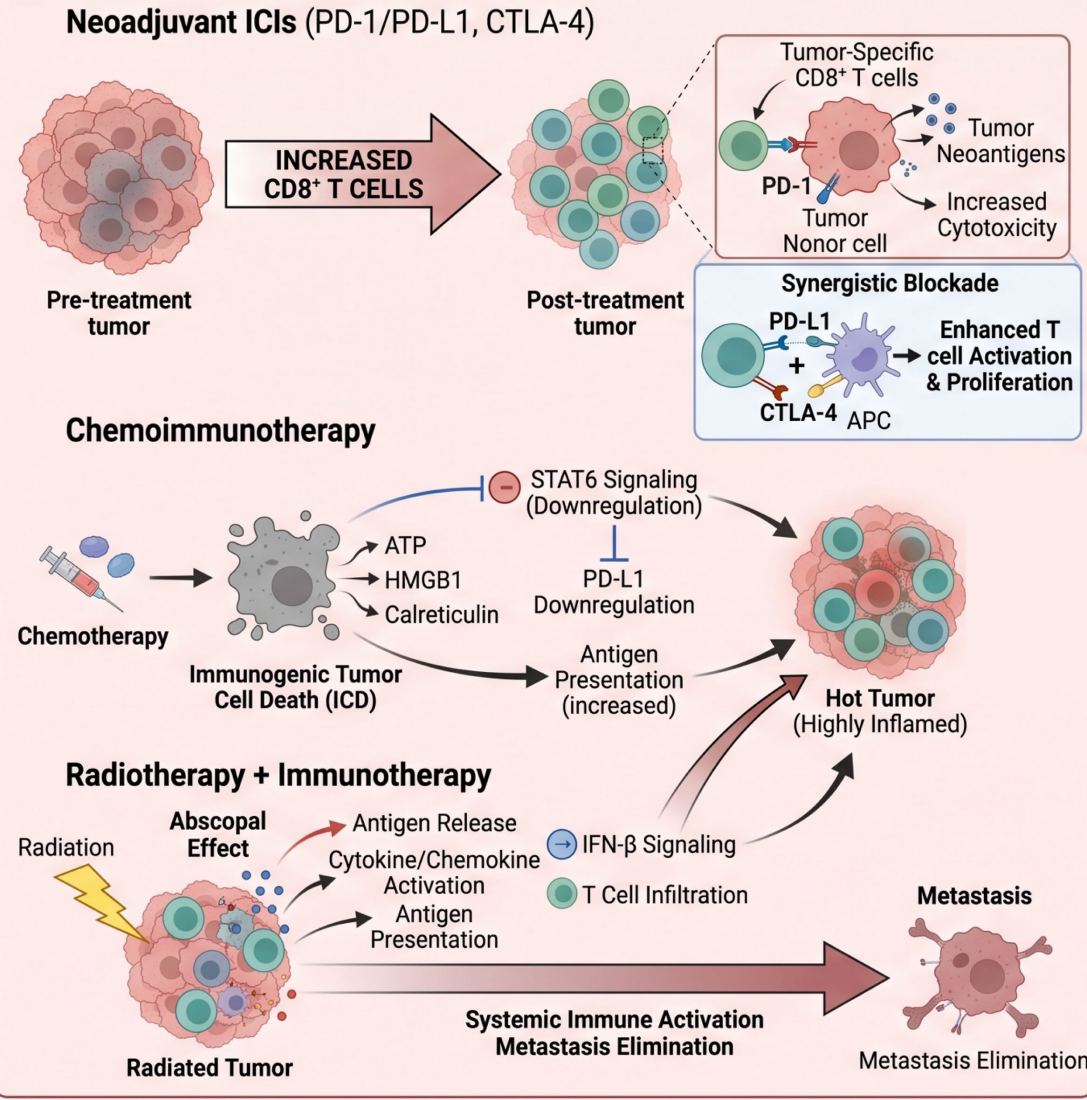
Neoadjuvant immunotherapy landscape in non-small cell lung cancer.

Chemoimmunotherapy can significantly improve progression-free survival (PFS) and overall survival (OS) in patients with advanced NSCLC (33, 34). These clinical observations support the mechanistic rationale that chemoimmunotherapy enhances anti-tumor immune responses. Chemotherapy suppresses tumor cell proliferation while exerting immunostimulatory effects. It modulates the activation of effector cells inhibited or activated during immune suppression, increases immune infiltration by innate and T cells, and promotes the expression of tumor antigens within the tumor microenvironment. These processes convert immunologically “cold” tumors with low detectable inflammation into “hot” tumors with significant inflammatory responses (35, 36). Several cytotoxic agents can induce immunogenic cell death (ICD), a regulated form of tumor cell death that couples direct cytotoxicity with immune activation (37). During ICD, dying tumor cells release or expose damage-associated molecular patterns (DAMPs), including high-mobility group box 1 (HMGB1), extracellular ATP, and calreticulin (38, 39). These signals promote dendritic cell recruitment, maturation, and antigen uptake, thereby strengthening tumor antigen processing and cross-presentation (40, 41). In parallel, chemotherapy may increase MHC-I expression on tumor cells and enhance the presentation of tumor-derived peptides, improving recognition by cytotoxic CD8^+^ T cells (42, 43). The release of ATP can further support inflammasome-associated inflammatory signaling, whereas HMGB1 promotes antigen-presenting cell activation through pattern-recognition receptor pathways (44). Chemotherapy may enhance responsiveness to PD-1/PD-L1 blockade by increasing antigen availability, reinforcing MHC-I–dependent antigen presentation, and promoting T-cell infiltration within the tumor microenvironment (45, 46) (**Figure 1**).

### 2.3 Principles of combination immunotherapy and radiotherapy

The integration of radiotherapy (RT) with immune checkpoint inhibitors (ICIs) represents a mechanistically grounded strategy to amplify systemic anti-tumor immunity in early-stage non-small cell lung cancer (NSCLC). This rationale is anchored in the abscopal effect—a phenomenon wherein localized radiation elicits systemic tumor regression at non-irradiated sites, mediated by the activation of adaptive immune responses (47, 48). Radiotherapy overcomes multiple tumor immune evasion mechanisms; for instance, it may release tumor-associated antigens, which activate inflammatory cytokines and chemokines to recruit immune cells. Additionally, radiotherapy can induce immune cell infiltration, increasing tumor susceptibility. These complex interactions help reprogram the tumor microenvironment and enhance the functionality of antigen-presenting cells and T cells (49).

RT remodels the tumor microenvironment (TME) through multiple complementary pathways. First, radiation-induced DNA damage and cellular stress upregulate type I interferon (IFN) signaling and pro-inflammatory cytokines (CXCL9/10), thereby enhancing the recruitment and activation of CD8^+^ T cells and natural killer (NK) cells (50–53). RT can reverse local immunosuppression by downregulating regulatory T cells (Tregs) and myeloid-derived suppressor cells (MDSCs) within the irradiated field, while concurrently increasing major histocompatibility complex class I (MHC-I) expression on tumor cells to improve antigen visibility (54–56). In addition, radiation-mediated vascular normalization may improve T-cell trafficking into the TME, further potentiating ICI efficacy (57–59). Collectively, these immunomodulatory effects convert immunologically “cold” tumors into “hot” phenotypes amenable to checkpoint blockade.

Clinical translation of this synergy has been substantiated in clinical trials (49, 60). In phase III, randomized, multicenter, international trials, MEDI4736 was evaluated as sequential therapy in patients with locally advanced, unresectable NSCLC (stage III). Patients with unresectable NSCLC received two-weekly durvalumab or placebo, for up to 1 year. Compared with placebo, durvalumab after concurrent chemoradiotherapy significantly extended PFS and OS (PFS: 1.5 years vs 0.5 years). Combining radiotherapy with immunotherapy reduced local recurrence and metastasis, enhancing systemic anti-tumor efficacy. Moreover, hypofractionated radiotherapy (6 Gy ×5 or 9 Gy ×3) combined with durvalumab was effective in advanced NSCLC, providing evidence for the abscopal effect. Radiation-induced antigen exposure increased systemic immune activation. Early dynamic changes in serum interferon-β levels and circulating T cell repertoires were identified as predictive factors for favorable responses to combined radiotherapy and immunotherapy (61, 62).

## 3 Current clinical trials

A growing number of clinical trials are investigating the efficacy of immunotherapy in the neoadjuvant setting for operable NSCLC. Early studies primarily evaluated single-agent immune checkpoint inhibitors (ICIs) (8, 63, 64), whereas recent investigations have expanded to neoadjuvant chemoimmunotherapy (65, 66), ICI combinations with anti-angiogenic agents (67), and multimodal regimens incorporating radiotherapy (67, 68). Most current evidence derives from small-scale, single-arm studies (8, 63, 67), with major pathological response (MPR) commonly adopted as a short-term efficacy endpoint (67, 69). Translational correlative studies frequently accompany these trials to elucidate underlying immunological mechanisms (70, 71). Nevertheless, definitive validation in early-stage NSCLC awaits results from large-scale, randomized, multicenter phase III studies.

### 3.1 Neoadjuvant immunotherapy and chemoimmunotherapy clinical trials

Emerging clinical evidence underscores the pivotal role of neoadjuvant immunotherapy within multimodal treatment paradigms for early-stage NSCLC. In the phase I LCMC3 trial, two cycles of neoadjuvant atezolizumab demonstrated acceptable safety in operable NSCLC, with 6% of patients experiencing grade ≥3 treatment-related adverse events (AEs) (64). Among enrolled patients, 88% proceeded to surgery; of these, 21% achieved MPR and 7% attained pathological complete response (pCR) within the primary efficacy population. MPR rates were notably higher in patients with PD-L1 expression ≥50% (33% vs. 11%; SP142 antibody), whereas tumor mutational burden (TMB) assessed by whole-exome sequencing showed only weak correlation with pathological regression. Tumors harboring STK11 mutations tended to exhibit diminished pathological response. Multiplex immunofluorescence analyses further revealed that baseline infiltration of CD68^+^ cells and CD3^+^/PD-1^+^ T cells was associated with improved MPR, suggesting that AI-assisted quantification may yield biologically and clinically relevant insights (53).

Neoadjuvant chemoimmunotherapy has demonstrated enhanced pathological responses and survival benefits in resectable NSCLC (34, 49). In a phase II trial, atezolizumab combined with carboplatin and albumin-bound paclitaxel yielded MPR and pCR rates of 57% and 33.3%, respectively, with a median disease-free survival (DFS) of 1.5 years (67). Notably, patients with EGFR or STK11 mutations rarely achieved pCR, highlighting the potential influence of driver alterations on treatment efficacy. Supporting the utility of ICI monotherapy, a study of two preoperative cycles of nivolumab reported an MPR rate of 45% without delaying surgical intervention (8). The NADIM trial evaluated neoadjuvant nivolumab plus chemotherapy followed by adjuvant nivolumab; at a median follow-up of 2 years, no treatment-related deaths or surgical delays occurred, and 85% of surgically treated patients remained alive and disease-free, with a 90% downstaging rate (72). In the phase II trial, neoadjuvant nivolumab plus ipilimumab achieved a higher MPR rate than nivolumab monotherapy (33% vs. 17%) in operable NSCLC (69). However, combination therapy was associated with increased toxicity: 60% of patients experienced treatment-related AEs, with grade ≥3 events occurring in 40% (combination) versus 20% (monotherapy), and disease progression during neoadjuvant therapy was observed in 7.5% of patients (73).

### 3.2 Neoadjuvant immunotherapy with ICIs in NSCLC

While PD-1 inhibitors are standard care for advanced NSCLC, their neoadjuvant application is emerging. NSCLC patients face high recurrence risks and suboptimal five-year survival following surgery alone, underscoring the need for effective, low-toxicity systemic therapies to eradicate micrometastases (74). Early-phase trials have established the safety and feasibility of short-course neoadjuvant PD-1 blockade. For instance, a phase I study demonstrated that two cycles of neoadjuvant PD-1 inhibition in stage IIA–IIIA NSCLC yielded favorable pathological responses without compromising surgical feasibility (74). Similarly, the clinical trial (NCT03030131) is evaluating complete resection following up to three cycles of neoadjuvant durvalumab in stage I–IIIA patients (75). Beyond multinational studies, domestic ICIs are showing promise. A recent investigation of neoadjuvant sintilimab (two cycles) in stage IA–IIB NSCLC reported an MPR of 41% and a pCR of 16%, with only 7.5% of patients experiencing surgical delays; post-treatment PET/CT scans confirmed tumor metabolic response (76). Currently, multiple trials are assessing other agents, including toripalimab (NCT04304248), carrelizumab (NCT04541251), camrelizumab (NCT03745222), tislelizumab (NCT03916627), and SHR-1316 (NCT04316364), alongside various combination regimens. Notably, the rapid expansion of chemoimmunotherapy trials compared to immunotherapy monotherapy reflects a strategic shift toward maximizing clinical benefit (67).

Current clinical trials are evaluating combinations of anti-angiogenic therapy with immunotherapy in the neoadjuvant setting, such as sintilimab plus chemotherapy (NCT03872661), pembrolizumab plus ipilimumab (NCT04040361), apatinib plus camrelizumab (NCT04506242). However, some trials are still recruiting. While combinations of anti-angiogenic therapy and immunotherapy have demonstrated efficacy in advanced NSCLC (77), their effectiveness in early-stage NSCLC remains unclear. Interim analyses from phase III trials of neoadjuvant chemoimmunotherapy and nivolumab in resectable NSCLC showed MPR of 78% and pCR of 39% (78). Similarly, randomized, single-center, phase II, open-label trials of neoadjuvant nivolumab monotherapy or combination with ipilimumab or radiotherapy have begun, designed for surgical resection in stage I–IIIA NSCLC (NCT03217071). Comparable studies of nivolumab plus durvalumab or nivolumab plus ipilimumab plus radiotherapy (anti-CTLA-4) are ongoing (**Supplementary Table 1**).

## 4 Predictive biomarkers of neoadjuvant immunotherapy in NSCLC

### 4.1 PD-L1 and tumor mutational burden

Robust predictive biomarkers are essential for optimizing patient selection and risk stratification in early-stage NSCLC immunotherapy (79). While PD-L1 expression and tumor mutational burden (TMB) are established predictors of response in advanced disease (80), their prognostic utility in the neoadjuvant setting remains inconclusive (81, 82). Although PD-L1 guides first-line therapeutic strategies in metastatic NSCLC, conflicting evidence challenges its applicability to early-stage disease. Some studies identify PD-1 expression as an independent prognostic factor for recurrence and mortality (83, 84); conversely, others report that PD-L1, TMB, effector T-cell infiltration, and interferon-γ signatures fail to predict overall survival (85), with no significant correlation observed between PD-L1 status and prognosis in resected cohorts (86). These discrepancies underscore the need for further validation of PD-L1 in perioperative protocols. TMB, reflecting tumor neoantigen load, represents a promising but complex biomarker. Although elevated TMB often correlates with improved immunotherapy responsiveness (87, 88), its clinical implementation is limited by heterogeneity. Notably, a TMB threshold >62 has been associated with poor prognosis following resection (89), whereas high blood-based TMB predicted superior progression-free survival with atezolizumab versus chemotherapy in pooled analyses (90). Despite these ambiguities, emerging data suggest that TMB may specifically predict major pathological response (MPR) in the neoadjuvant setting (81, 82), warranting standardized assessment frameworks (91, 92).

### 4.2 Circulating tumor DNA

Advances in liquid biopsy have positioned circulating tumor DNA (ctDNA) as a dynamic, non-invasive biomarker for monitoring therapeutic responses in NSCLC. Derived from apoptotic or necrotic tumor cells, ctDNA is detectable in peripheral blood and reflects real-time tumor genomic status (93, 94). In the neoadjuvant setting, ctDNA analysis offers significant potential for risk stratification and early assessment of treatment efficacy (95, 96). Preoperative ctDNA levels have been shown to predict disease-free survival (DFS) and overall survival (OS) following complete resection in locally advanced NSCLC, supporting its utility in guiding perioperative therapeutic decisions (97). Furthermore, ctDNA clearance during or after neoadjuvant therapy may serve as a surrogate endpoint for long-term DFS and OS (98), a concept currently under prospective evaluation in trials such as NCT04367311, which integrates ctDNA monitoring with neoadjuvant atezolizumab and chemotherapy. Parallel evidence from breast cancer demonstrates that ctDNA dynamics precisely reflect residual disease and pathological response post-neoadjuvant therapy (98), suggesting analogous applications in NSCLC. As assay sensitivity improves and standardization advances, ctDNA is poised to become a cornerstone biomarker for personalizing neoadjuvant immunotherapy strategies.

### 4.3 Other potential biomarkers

Accessible hematological parameters, including neutrophil-to-lymphocyte ratio (NLR), platelet-to-lymphocyte ratio (PLR), and lymphocyte subset profiling, may offer prognostic insights. Established serum biomarkers such as carcinoembryonic antigen (CEA) and CYFRA 21–1 have long facilitated treatment monitoring (99); meta-analyses confirm CYFRA 21–1 tracks chemotherapy response, suggesting potential utility in predicting neoadjuvant ICI efficacy (100). The immune microenvironment undergoes substantial remodeling during therapy: comparative analyses of resected tumors versus adjacent normal tissue indicate that baseline immune profiling can stratify postoperative outcomes (100), implicating the overall immune landscape as a composite biomarker. Emerging evidence also links gut and pulmonary microbiome composition to ICI responsiveness, potentially modulating therapeutic efficacy through immune regulation (101). Future investigations should systematically characterize pre- and post-neoadjuvant microenvironmental dynamics, including microbiota shifts, semi-quantitative lactoferrin-3 expression, and CD8^+^ T-cell infiltration patterns (102). Additionally, spatial analysis of T-cell receptor clonality has been proposed as a predictive metric for pathological response to neoadjuvant anti-PD-1 therapy in resectable NSCLC (103), while peripheral TCR sequencing may further refine response prediction. Integrating these multimodal biomarkers could enhance precision in patient selection and therapeutic monitoring.

## 5 Conclusion

Neoadjuvant immunotherapy has emerged as a promising strategy to enhance treatment outcomes in early-stage NSCLC. ICIs, alone or in combination with chemotherapy, radiotherapy, or dual checkpoint blockade, can potentiate anti-tumor immune responses, reduce micrometastatic disease, and improve long-term survival. Preclinical models demonstrate that these therapies increase infiltration of tumor-specific CD8^+^ T cells, modulate the tumor microenvironment, and overcome immune suppression, leading to improved pathological responses. Clinical trials have further demonstrated the feasibility and potential efficacy of neoadjuvant immunotherapy, with encouraging major pathological response and pathological complete response rates.

Nevertheless, several challenges must be addressed before this strategy can be broadly optimized in clinical practice. First, immune-related adverse events may delay surgery, require corticosteroid or immunosuppressive treatment, and complicate perioperative recovery, emphasizing the need for multidisciplinary monitoring and timely toxicity management. Second, intrinsic or acquired resistance remains a major limitation. In particular, STK11 and KEAP1 mutations may define an immune-refractory phenotype characterized by impaired T-cell infiltration, altered oxidative stress regulation, metabolic adaptation, and reduced responsiveness to PD-1/PD-L1 blockade. Third, standardized biomarkers for patient selection and response prediction remain lacking. Although PD-L1 expression, tumor mutational burden, circulating tumor DNA, and immune profiling have shown potential value, their predictive performance varies across studies because of differences in assay platforms, cut-off values, sampling time points, and endpoint definitions. Future research should therefore prioritize large randomized trials, harmonized biomarker frameworks, and mechanism-informed combination strategies to improve efficacy while preserving surgical feasibility and patient safety.
